# Perchlorates in the treatment of hyperthyroidism and thyrotoxicosis: a comprehensive review

**DOI:** 10.1007/s12020-023-03679-y

**Published:** 2024-01-09

**Authors:** Giuseppe Lisco, Giacomo Accardo, Cinzia Pupilli, Pasqualino Malandrino, Vincenzo De Geronimo, Vincenzo Triggiani

**Affiliations:** 1 Interdisciplinary Department of Medicine, School of Medicine, University of Bari “Aldo Moro”, Bari, 70124 Italy; 2 Dipartmento di Scienze Mediche, Chirurgiche, Neurologiche, Metabolismo ed Invecchiamento, Università degli Studi della Campania “L. Vanvitelli”, Napoli, 80133 Italia; 3 SOSD Endocrinologia – Azienda USL Toscana Centro, Firenze, 50122 Italia; 4 Endocrinologia, Dipartimento di Medicina Clinica e Sperimentale, Arnas Garibaldi, Università di Catania”, Catania, Italy; 5 Servizio di Endocrinologia, Morgagni CCD, Catania, 95125 Italia

**Keywords:** Perchlorates, Thyrotoxicosis, Iodine media-contrast hyperthyroidisms, Amiodarone-induced hyperthyroidisms, Hyperthyroidism in pregnancy, Thyroid storm

## Abstract

**Introduction:**

Perchlorates are ionic inhibitors antagonizing iodine transport into thyrocytes, hampering thyroid hormone synthesis. Nevertheless, perchlorates are not considered as first-line treatment in hyperthyroidism and thyrotoxicosis as compared to other pharmacological and non-pharmacological interventions.

**Aim:**

Reassessing the therapeutic role of perchlorates in hyperthyroidism and thyrotoxicosis throughout a systematic review of the Literature.

**Methods:**

Guidelines were searched and examined to summarize current recommendations on the use of perchlorates in the management of hyperthyroidism. Randomized and non-randomized clinical trials were also searched and reviewed to summarize the efficacy/effectiveness and safety of perchlorates in hyperthyroidisms and thyrotoxicosis.

**Results:**

The management of specific forms of hyperthyroidism was considered, including Graves’ disease (GD) in non-pregnant adults, hyperthyroidisms in pregnancy, iodine media contrast-induced hyperthyroidism, amiodarone-induced hyperthyroidisms, and thyroid storm. Most of the reported studies had remarkable limitations in terms of study design (non-controlled trials, lack of blinding), low number of participants, and the lack of clinically relevant endpoints, such as cardiovascular events, cardiovascular mortality, and teratogenicity. Overall, perchlorates could be considered a second-line treatment after thionamides, radioiodine, and total thyroidectomy in both GD and hyperthyroidisms in pregnancy. The therapeutic potential of perchlorates alone or in combination with other agents could be considered a second-line treatment of iodine-related hyperthyroidisms and thyroid storm.

**Conclusion:**

Despite the low level of evidence, perchlorates could be considered in such specific forms of thyroid disorders, including iodine-induced hyperthyroidism and thyroid storm.

## Introduction

Perchlorates naturally occur in the atmosphere due to a photogenic reaction between elementary Chlore and Ozone in the stratosphere. Alternatively, they originate from industrial manufacturing products of fertilizers, explosives, propellants, and disinfectants [1].

The mechanisms by which perchlorates affect thyroid function and the therapeutical potential of perchlorates in thyroid dysfunctions have been well-known since the 1950^th^ [2]. Perchlorates compete with the iodine transport at the basal-lateral membrane site of thyrocytes. The Sodium (Natrium)-Iodine Symporter (NIS), a 13-domain transmembrane channel acting as a secondary active transporter thanks to the persisting action of the sodium-potassium ATPase, here regulates iodine entry into thyrocytes by exchanging it with two sodium ions [3].

Sufficient iodine supply is critical for thyroid hormone synthesis. Once iodine is consumed with food intake, it is absorbed at the small intestinal site. The 95% of the entire iodine pool is then internalized into thyrocytes. Acute and chronic overexposure to perchlorates reduces the efficiency of iodine transport, as perchlorates compete with iodine internalization into thyrocytes at the NIS site. The drop of intrathyroidal iodine transport transmutes into a reduced intrathyroidal concentration of the ion with a consequent reduction of the halogenation of tyrosine residues in the thyroglobulin. Thanks to this mechanism, perchlorates could be considered in the treatment of specific forms of hyperthyroidisms, which are characterized by exaggerated exposure to radioactive iodine as the consequence of diagnostic/therapeutic procedures or nuclear disasters. On the other hand, chronic exposure to perchlorates is associated with a relevant risk of multinodular goiter and differentiated thyroid cancer [4].

## Pharmacological characteristics of perchlorates

Perchlorates have a short half-life after oral administration. Plasmatic and intrathyroidal concentrations of perchlorates peak respectively a few minutes and around 4 h after the administration. The therapeutic effect of a single dose of perchlorates lasts longer than 8 h, leading to repeated administration over 24 h to obtain a consistent pharmacological effect (3 to 4 times a day). The pharmacological effect of perchlorates depends on the circulating perchlorate-to-iodine ratio, background hyperthyroidism severity, and background treatments (such as thionamides).

Perchlorates do not require specific catabolism and are usually excreted with urine (renal clearance >95%).

Potassium perchlorate was available in hard-gel capsules 200 mg. The starting dose was 200 mg (1 tablet) three times a day (600 mg) with subsequent titration according to the clinical response of hyperthyroidism after four weeks of treatment. The effective total daily dose of potassium perchlorates did not exceed 1,000 mg. Potassium perchlorate is no longer available in Italy since 2013 when it was entirely replaced by sodium perchlorate.

Sodium perchlorate is available as oral drops (344 mg for each mL solution). The effective daily dose is 800–1000 mg (10 drops, 4 to 5 times daily).

Perchlorates are usually well-tolerated. Adverse effects include dermal rash, nausea, weakness, oral and pharyngeal dryness, leukopenia, and lymph node enlargement (Table 1). Gastrointestinal signs and symptoms, such as nausea, vomiting, abdominal pain, and icterus, usually indicate overexposure to perchlorates (https://farmaci.agenziafarmaco.gov.it/aifa/servlet/PdfDownloadServlet?pdfFileName=footer_000194_013346_RCP.pdf&retry=0&sys=m0b1l3, https://ansm.sante.fr/uploads/2020/12/09/38386213e9b0426ba4f5929608ea4e52.pdf).

**Table 1:** Summary of adverse events associated with the therapeutic assumption of perchlorates. Clinical manifestations are grouped in order of frequency (common, non-common, rare)

Common (≥1%–<10%)	Rash
	Nausea or vomiting
	Oral mucosal dryness
	Pharyngitis
	Lymph node enlargement
	Leukopenia
	Purpura
	Fever and arthralgias
Ucommon (≥0.1%–<1%)	Diarrhea
	Myalgias
	Paresthesia
	Headache
	Eosinophilia
	Itching
	Icterus
	Agranulocytosis
Rare (<0.01%)	Thrombocytopenia/Aplastic anemia
	Albuminuria
	Nephrotic syndrome
	Alopecia
	Acne
	Dermatitis
	Urticaria
	Liver toxicity
	Erythema nodosum
	Antinuclear antibodies and anti-erythrocyte antibodies
	Duodenal ulcer perforation

## Hyperthyroidism and thyrotoxicosis

Hyperthyroidism indicates the presence of pathologically high levels of circulating thyroid hormones due to uncontrolled thyroid production, while thyrotoxicosis is characterized by metabolic and hemodynamic changes due to systemic overexposure to thyroid hormones [5].

The most common forms of hyperthyroidism include GD (60-80%), multinodular toxic goiter (15–20%), and autonomously functioning thyroid adenoma (<5%). Thyrotoxicosis can be the consequence of hyperthyroidism or not. In the latter case, levothyroxine overdose in patients on thyroid replacement therapy is the most common cause of thyrotoxicosis. Subacute, silent, and postpartum thyroiditis are the other common causes of thyrotoxicosis not attributable to hyperthyroidism. Thyrotoxicosis without hyperthyroidism is a self-limiting condition requiring symptomatic treatment to attenuate specific disturbs such as tachycardia, anxiety, and insomnia.

Thyroid storm is a rare but severe form of thyrotoxicosis that is considered an endocrine emergency with a mortality rate of around 20%. Thyroid surgery, anesthesia, stress, and radioiodine therapy may induce thyroid storm in predisposed individuals.

The most common clinical presentation includes hyperthermia, associated with severe thermodynamic dysfunction, tachyarrhythmias, heart failure, nausea, vomiting, diarrhea, icterus, psychosis, seizures, and coma [5].

Acute and chronic iodine exposure leads to adverse thyroid effects, including a complete saturation of the NIS and the enzymatic machinery involved in thyroid hormone synthesis. This effect was first detected and described by Wolff and Chaikoff [6], indicating the existence of a protective mechanism against iodine-induced overload and oxidative stress in the thyroid gland. The so-called Wolff-Chaikoff effect is usually transient, but patients with certain thyroid diseases, such as Hashimoto’s thyroiditis, are at risk of occurring or relapsing hypothyroidism due to a persisting block of thyroid hormonogenesis [7]. Less frequently, iodine overexposure induces hyperthyroidism, especially in older patients with inveterate multinodular goiter or in those with euthyroid or subclinical thyroid autonomy, chronic and severe iodine deficiency, and GD. The mechanism underlying iodine-induced hyperthyroidism is also known as the Jod-Basedow phenomenon. Iodine media contrast [8] and type 1 amiodarone-induced (AIT) hyperthyroidisms [9] are the most common clinical examples of iodine-induced hyperthyroidisms.

Iodate media contrast-induced hyperthyroidism is a rare complication of iodine overexposure in the short term [10, 11], even if the long-term risk of thyroid dysfunction is more than doubled for hyperthyroidism and more than triplicated for hypothyroidism [12]. Other risk factors of iodate media contrast-induced hyperthyroidism are repeated exposure to iodine media contrast and the presence of thyroidal predisposing factors, such as euthyroid multinodular goiter. It has been estimated that the overall prevalence of iodine media contrast-induced hyperthyroidism is less than 15%, with relevant variability based on the level of iodine exposure of the examined population [13].

## Guidelines on the management of hyperthyroidism and thyrotoxicosis: general aspects and focus on ionic inhibitors

The management of hyperthyroidisms includes the use of pharmacological (antithyroid medications) and non-pharmacological interventions, such as radioactive iodine and thyroid surgery. Symptomatic treatment with beta-blockers is indicated to alleviate signs and symptoms of thyrotoxicosis, mainly high rest heart rate (e.g., >90 beats per minute). Ionic inhibitors can be considered among the plethora of pharmacological interventions in the following conditions: prevention of radioactive iodine-induced thyrotoxicosis (Recommendation 7, American Thyroid Association – ATA 2016); in combination with antithyroid medications (such as methimazole) to prevent potential adverse events in patients expected to require high-dose regimen (e.g., more than 30 mg/day) (Recommendation 14, ATA 2016); peri-surgical management of hyperthyroidism in preparation to thyroidectomy (Recommendation 24 and 26, ATA 2016); background treatment of thyroid storm (Recommendation 35, ATA 2016); as alternative treatment when antithyroid medications, radioactive iodine, and thyroid surgery are not tolerated or contraindicated (Recommendation 36, ATA 2016) [5].

According to the 2018 European Thyroid Association (ETA) Guidelines for GD, ionic inhibitors should be considered in the perioperative management of candidate adult patients to total thyroidectomy (Recommendation 27, ETA 2018) [14] and pediatric patients [15].

Both the ATA and ETA Guidelines did not mention perchlorates among ionic inhibitors. Instead, the task forces mentioned the potassium iodide (ATA) and the highly concentrated (1 g/mL) saturated solution of potassium iodide (ATA and ETA) as the only ionic inhibitors suitable for clinical use (https://dailymed.nlm.nih.gov/dailymed/fda/fdaDrugXsl.cfm?setid=ca1d3449-ea29-49a4-8863-365ec95f1553&type=display).

Less solid evidence suggests that ionic inhibitors can be considered an alternative therapeutic option during the first trimester of pregnancy, alone or in combination with ion-exchange resins, such as cholestyramine, that are also approved for intrahepatic cholestasis of pregnancy [16]. These specific guidelines (ATA 2017) mentioned sodium perchlorate among the ionic inhibitors, indicating that ionic inhibitors are effective and safe (no described teratogenic effects compared to lithium carbonate) in maintaining euthyroidism in pregnancy (Recommendation 45, ATA 2017).

The 2018 ETA Guidelines on the management of amiodarone-induced thyroid dysfunction mentioned perchlorates as candidate pharmacological treatment in combination with methimazole in type 1 AIT (Recommendation 5) and glucocorticoids in type 2 AIT (Recommendation 6, 7, and 8).

Type 2 AIT is a self-limiting thyrotoxicosis not requiring amiodarone to be discontinued. Given the clinical presentation and evolution of type 2 AIT, ionic inhibitors could be considered as a therapeutic alternative for optimizing recovery from thyrotoxicosis and avoiding unnecessary use of a high-dose methimazole regimen. In this case, the total daily dose of sodium perchlorate should be at most 1000 mg, up to no more than six weeks [17].

Iodine concentration in the most commonly available media contrasts ranges from 180 to 370 mg/mL. It means that the overall amount of iodine administered when carrying out diagnostic radiological examination ranges from 18 to 45 grams, depending on the patient’s characteristics (i.e., body weight), range of radiological scan, and the size of anatomical sections to be scanned [18]. As the recommended daily allowance of iodine is no more than 300 μg, acute exposure to iodinated contrast media equals an overload dose by 60–150 times higher than recommended. Overt hyperthyroidism usually contraindicates radiological diagnostic testing with iodine media contrasts when deferable. Nevertheless, in the case of urgent and non-deferrable radiological procedures, the 2021 ETA Guidelines suggest intensifying the pharmacological management of hyperthyroidism to prevent any deterioration of thyroid dysfunction after the exposure to iodine media contrast (Recommendation 2), particularly in old patients or in those at high or very high cardiovascular risk [19]. Prophylactic pharmacological intervention is not routinely recommended to prevent iodine media contrast-induced hyperthyroidism in all individuals. Conversely, the treatment should be individualized based on risk factors [19].

Ionic inhibitors, including perchlorates, can be considered as a therapeutic option, in combination with methimazole, for treating iodinated contrast media-induced hyperthyroidism at the maximum daily dose of 1,000 mg up to 4 weeks and for prophylaxis of iodine media contrast-induced hyperthyroidism at 1,000 mg per day up to 1-2 weeks (Recommendation 9 and 21, ETA 2021). The summary of the main recommendations is listed in Table 2.

**Table 2:** Summary of recommendations from examined Guidelines on the use of ionic inhibitors in the treatment of hyperthyroidisms and thyrotoxicosis

Guidelines	Recommendations	Clinical indications	Ionic inhibitor
ATA 2016	7, strong, low level of evidence (section D1)	Treatment of hyperthyroidism before (or soon after) radioactive iodine	Potassium iodide, saturated solution of potassium iodide
ATA 2016	14, strong, low level of evidence (section E1)	Thyrotoxicosis in combination to thionamides for reducing the risk of thionamide-related adverse events	Potassium iodide, saturated solution of potassium iodide
ATA 2016	24 e 26, strong, low level of evidence	Perioperative management of thyrotoxicosis ahead of thyroid surgery	Potassium iodide, saturated solution of potassium iodide
ATA 2016	35, strong, low level of evidence	Multimodal treatment of thyroid storm	Potassium iodide, saturated solution of potassium iodide
ATA 2016	36, not recommended, insufficient level of evidence	Treatment of GD when thionamides, radioactive iodine, and thyroid surgery are contraindicated or not tolerated	Potassium iodide, saturated solution of potassium iodide
ATA 2017	45, strong, high level of evidence	Hyperthyroidism in pregnancy as alternative to propylthiouracil	Potassium iodide
ETA 2018 Graves	27, weak, moderate level of evidence	Perioperative management of GD ahead of thyroid surgery (10 days before)	Saturated solution of potassium iodine
ETA 2018 Amiodarone	5, strong, moderate level of evidence	Treatment of type 1 AIT	Sodium perchlorate
ETA 2018 Amiodarone	6, strong, moderate level of evidence 7, weak, low level of evidence 8, weak, low level of evidence	Potential for type 2 AIT in combination to thionamides and glucocorticoids	Sodium perchlorate
ETA 2021	2, weak, low level of evidence	Prophylaxis of iodine media contrast induced hyperthyroidism in at-risk patients (existing thyroid dysfunction, advanced age, high cardiovascular risk)	Sodium perchlorate
ETA 2021	9, weak, low level of evidence 21 strong, very-low level of evidence	Prophylaxis of iodine media contrast induced hyperthyroidism in at-risk patients (existing thyroid dysfunction, advanced age, high cardiovascular risk) in combination to thionamides	Sodium perchlorate

*ATA* American Thyroid Association, *ETA* European Thyroid Association, *AIT* Amiodarone Induced hyperThyroidism

## Evidence on the use of perchlorates in hyperthyroidism and thyrotoxicosis

### Prophylaxis and treatment of iodinated contrast media-induced hyperthyroidism

Intervention trials assessing the efficacy/effectiveness and safety of perchlorates in this clinical setting were mainly conducted in Central Europe. One randomized clinical trial, published in 1996, was completed on 51 patients with euthyroid autonomously functioning nodules (pre-toxic phase) who underwent coronary angiography. These patients represented a population subgroup of particular interest. They were at high risk of iodine media contrast-induced hyperthyroidism following non-deferable procedures in a clinical contest of high cardiovascular risk with the need for limiting the chance of occurring hyperthyroidism in the short and long term. Patients were randomized to receive methimazole 20 mg/day (*n* = 17), sodium perchlorate (*n* = 17), and control (no treatment, *n* = 17). The intervention lasted 16 days (from one day before coronary angiography to 14 days after the procedure) [20]. Both active treatment groups showed similar thyrotropin (TSH) values, thyroid iodine uptake (scintigraphy), and 24-h urinary iodine excretion by comparing time zero with 30 days after. Controls exhibited a relevant reduction of TSH values with a relevant reduction of thyroid iodine uptake and 24-h urinary iodine excretion. Four cases of hyperthyroidism were described (2 controls and one case for each arm of treatment). Although the study results elucidated several physiological mechanisms related to the potential benefit of antithyroid treatment to prevent iodinated contrast media-induced hyperthyroidism, the clinical soundness of the findings was far from being acceptable for supporting extensive use of antithyroid treatment in this cluster of patients.

One non-randomized observational study was conducted on 32 patients with euthyroid multinodular goiter exposed to iodinate media contrast following computerized tomography, coronary or peripheral angiography, or transcatheter aortic valvuloplasty. Among them, 12 were allocated to receive prophylactic intervention with methimazole alone or in combination with ionic inhibitors (including perchlorates); the remaining did not undergo prophylaxis [21]. Methimazole (20 to 40 mg per day) was administered for 16 days (from one day before to 14 days after the procedures). Potassium perchlorate was administered at 900 mg daily up to 10 days after the procedures. The follow-up visits were carried out 4 to 405 days after administering iodinated contrast media. Seventeen patients underwent at least two administrations of iodinated contrast media during the follow-up. Twenty-one patients (58%) were diagnosed with hyperthyroidism, with a remarkable difference in the frequency of diagnosis between the two groups (15% prophylaxis vs. 65% non-prophylaxis, *p* < 0.01). The evolution of media contrast-induced hyperthyroidism averaged 52 days. No cases of overt hyperthyroidism were diagnosed in the group of patients treated with antithyroid medications. Overall, the results of this study demonstrated the therapeutic effectiveness of prophylactic treatment with antithyroid medication in attenuating the risk of long-term incident iodine media contrast-induced hyperthyroidism. Nevertheless, the lack of randomization and specific cardiovascular endpoints significantly reduced the evidence level for recommendation.

A third study has recently been published to reassess the relevance of the prophylaxis of iodine media contrast-induced in high-risk patients: people aged more than 65 years; admission to the coronary unit because of coronary revascularization or valvuloplasty; thyroid diseases predisposing to iodine media contrast-induced hyperthyroidism (GD, multinodular goiter, autonomously functioning adenoma; overt or subclinical hyperthyroidism). Patients (*n* = 61, mean age 72 years) were allocated in two groups: prophylaxis with methimazole alone or in combination with sodium perchlorate (*n* = 23) and no treatment (*n* = 38). The mean daily methimazole dose was 10 mg when it was administered alone (5 patients) and in combination with sodium perchlorate (*n* = 17) as well. Only one patient received sodium perchlorate alone. The mean daily perchlorate dose was 600 mg, administered from 7 to 14 days. The combined prophylaxis with methimazole and perchlorate was administered for ten days in the mean. It is unclear the duration of prophylaxis with methimazole alone. Methimazole was administered in 3 naïve patients before the study entry; the starting dose of methimazole was based on background levels of free thyroxine (fT4) and free triiodothyronine (fT3), also considering individual comorbidities. The clinicians involved in the study discretionally selected the candidate patients for prophylaxis, what kind of therapeutic regimen would have been appropriate for each patient (perchlorate alone, methimazole alone, or combination) and total daily dose, and the duration of prophylaxis. Thyroid function was checked at baseline, 4–10 days, 11–30 days, and 30–90 days after the procedures. Patients who received prophylaxis compared to controls had lower baseline body mass index and TSH and higher fT4 values. Thyroid function 11 to 30 days after the procedures was unchanged in 58%, improved in 28%, and worsened in 15% of patients. Risk factors associated with occurring hyperthyroidism were baseline impaired renal function (creatinine 1.14 mg/mL in mean), higher TSH values (1.13 mUI/L), and no prophylaxis (8 cases, 21% vs. 1 case, 4%). One fatality was also registered. The patient had multinodular goiter and background overt hyperthyroidism, and was admitted to the coronary unit for revascularization while on methimazole and sodium perchlorate two days before the angiography. Death was attributable to the deterioration of background cardiovascular disease. Lastly, the authors presented a cost estimation of prophylaxis. Prophylaxis with methimazole costs around 3.48 euros, while prophylaxis with methimazole and sodium perchlorate costs 7.91 euros per person [22]. Although this study was well-designed and conducted, it did not resolve criticisms highlighted by previous studies. More precisely, the lack of randomization, the low number of participants and outcomes, the lack of cardiovascular endpoints, and the relatively low follow-up limited the level of evidence of the results. Moreover, there were considerable selection and allocation biases due to patients’ selection, medications to use, titration, and different evolution of prophylaxis.

### Treatment of amiodarone-induced hyperthyroidisms

A pilot study published in 2003 showed that a stepwise approach based on the administration of methimazole and potassium perchlorate for 30 days, followed by glucocorticoids in patients not achieving euthyroidism due to persisting or relapsing hyperthyroidism after an initial response, was associated with better outcomes in AIT. The stepwise approach overcomes the therapeutic uncertainty of the management of AIT. On the one hand, it could be challenging to establish the leading mechanism of hyperthyroidism to differentiate type 1 from type 2 AIT, considering that the overlapping form of AIT is frequently observed. On the other hand, the stepwise approach restricts the use of glucocorticoids in terms of number of treated patients and cumulative dose-exposure. More precisely, the study found that starting with methimazole (30–50 mg/day) and potassium perchlorate 1,000 mg/die up to 30 days resulted in the restoration of euthyroidism in 12 of 20 patients (7 initially diagnosed with type 1 and 5 with type 2 AIT). Therefore, only eight patients received oral glucocorticoids (prednisone 40–80 mg/day) as they did not respond to methimazole and perchlorates. All patients achieved euthyroidism at the end of the trial [23].

No additional randomized controlled trials were conducted to confirm or deny the efficacy of this approach in AIT, so the lack of solid evidence in the field has generated relevant discrepancies in the clinical management of AIT worldwide. An international survey published in 2008 highlighted differences and analogies between American and European Endocrinologists. According to the survey’s responders, AIT was the most prevalent thyroid dysfunction among amiodarone users in Europe but not in the United States (hypothyroidism was more prevalent compared to hyperthyroidism). European, compared to American Endocrinologists, performed a comprehensive diagnostic workup while diagnosing AIT, including concomitant measurement of TSH, fT4, fT3 values, thyroid autoimmunity, neck ultrasound with color Doppler examination, and thyroid scintigraphy. More than European, American Endocrinologists declared that amiodarone discontinuation was usually unnecessary in type 1 and, more frequently, in type 2 AIT. Thionamides were the treatment of choice for type 1 AIT. Combining methimazole with perchlorates was more commonly observed in Europe than in the United States (31% vs. 15%), and the same was true for the combination of methimazole plus glucocorticoids in type 2 AIT [24].

One Dutch randomized, unblinded, controlled trial (2012) explored the efficacy and safety of sodium perchlorate in type 2 AIT [25]. The pharmacologic rationale was in the potential of sodium perchlorate to mitigate the cytotoxic effect of amiodarone on thyrocytes. All the participants were diagnosed with type 2 AIT and were advised not to discontinue amiodarone. All patients received methimazole 30 mg per day over the entire study duration. Besides background methimazole treatment, they were randomized to receive prednisone 30 mg/day (*n* = 12, group 1), sodium perchlorate 1,000 mg/day (*n* = 14, group 2), or both perchlorate and glucocorticoids (*n* = 10, group 3). The study endpoint was the achievement of euthyroidism, defined as a TSH value of more than 0.4 mUI/L while on amiodarone. Follow-up visits were scheduled every four weeks within the first six months, bimonthly from 6 to 12 months, and every six months during the second year. TSH was checked before each follow-up visit to manage medical treatment as follows: 1) if TSH was equal to or more than 0.4 mUI/L, the treatment was tapered until discontinuation in two weeks; 2) if TSH was lower than 0.4 mUI/L after three months from the randomization, the treatment was intensified by adding sodium perchlorate 1,000 mg/day (if group 1) or prednisone 60 mg/day (if group 2); 3) if TSH was <0.4 mUI/L after six months from the randomization, the treatment was furtherly potentiate at discretion of clinicians. Patients were mostly men, with a mean age lower than 60 years, and a mean TSH of 0.01 mUI/L. Group 1 and group 3 patients achieved euthyroidism, while prednisone 30 mg/day was added to background treatment in four (29%) patients of group 2 (who were on sodium perchlorate only). Time to normalization of TSH was shorter in group 1 (4 to 20 weeks, 8 on average) than in group 2 (4 to 32 weeks, 14 on average) and group 3 (4 to 28 weeks, 12 on average). A similar trend was also observed for fT4: group 1 (4 weeks in mean), group 2 (12 weeks in mean), group 3 (8 weeks in mean). None discontinued amiodarone. One relapsing hyperthyroidism was documented in group 1 (after 24 weeks). It was managed easily with the achievement of euthyroidism in 8 weeks. Two cases of relapsing hyperthyroidism were detected in group 3 after 12 and 72 weeks, respectively (remission in 4 weeks for both). Most participants reached normal TSH values at the end of the follow-up. Two participants from group 1, one from group 2, and three from group 3 were diagnosed with hypothyroidism (TSH > 5 mUI/L) and were consequently replaced with levothyroxine. One patient of group 1 with an established diagnosis of type 2 diabetes on a background insulin regimen experienced relevant deterioration of glucose control, which was managed by increasing the mean daily insulin dose. One patient of group 2 had myalgias accompanied by serological elevation of creatine phosphokinase and dermal exanthema after two weeks of treatment. The other two patients in the group 2 had nausea. One patient of the group 3 had serological elevation of transaminase after 12 weeks of treatment. Two patients of group 2 did not complete the study: one died after around 18 months of follow-up, and the second underwent cardiac transplantation after 21 weeks from the randomization. Moreover, one patient of the group 3 did not complete the follow-up due to methimazole-induced agranulocytosis while treating relapsing thyrotoxicosis. Overall, the study results indicate that sodium perchlorate alone is less effective than prednisolone when added to methimazole in treating type 2 AIT. This study has the same limitations mentioned above.

### Treatment of Graves’ disease

Evidence suggests that thionamides, compared to potassium perchlorates, are similarly effective in GD [26], thus confirming the remission of GD increases along with the decline of anti-TSH receptor antibody title over the follow-up but controverting an immunomodulatory adjuvant effect of thionamides [26, 27]. Nevertheless, thionamides are the treatment of choice of GD [28]. Thionamides are potent antithyroid medications to restore euthyroidism upon the treatment has started, despite their unpredictable effect on the risk of relapsing/recurring hyperthyroidism. The daily dose of thionamides depends on the severity of hyperthyroidism, as the antithyroid effect of thionamides is dose-dependent. It is unclear whether adverse events related to chronic exposure to thionamides are entirely dose-dependent since severe adverse events have also been described in patients who had assumed a low-moderate daily dose regimen.

Ionic inhibitors, including saturated iodide solutions, were more frequently prescribed in the “pre-thioamides era” [29]. They could be considered as a second-choice treatment of GD. One Japanese observational study assessed the effectiveness of potassium iodide in patients with GD who had developed intolerance or contradictions to thionamides (*n* = 204) [30]. Among these 204 patients, 44 received potassium iodide, and 29 (66%) achieved adequate control of thyroid hormone levels with a mean daily dose ranging from 10 to 400 mg. Seventeen individuals (40%) achieved the euthyroidism through the entire 7-year follow-up. Fifteen individuals did not achieve the euthyroidism, although a high-dose potassium iodide (up to 750 mg/day) was prescribed. Seven of them controlled the disease after the reintroduction of low-dose methimazole. Around 70% of participants who were assuming potassium iodide (<200 mg/die) obtained adequate control of GD over the 7-year follow-up. Only 35% of patients who had taken high-dose iodine (>200 mg/die) achieved euthyroidism. Overall, the result of this study indicates that ionic inhibitors can be an effective pharmacological alternative in patients with hyperthyroidism for whom thionamides are contraindicated or not tolerated. However, it should be considered that the chance of remission of GD was significantly lower in patients requiring a high-dose potassium iodide regimen (i.e., >200 mg/day). The low number of participants, the observational design of the study, the lack of randomization, and the management of patients during the follow-up lead to considerable selection and allocation biases and reduce the level of evidence of results.

Two observational studies conducted in patients with GD who underwent thyroidectomy showed that the administration of iodate solution (Lugol solution, potassium iodide 100 mg/mL) 10 days before surgery, compared to no treatment, significantly reduced thyroid vascularization, intrathyroidal inflammation and suppressed effectively thyroid hormone synthesis [31, 32]. The approach is also associated with faster recovery from hyperthyroidism ahead of thyroid surgery and lower risk of intraoperative bleeding, thus increasing the level of safety of the entire surgical procedure. The ETA guidelines have implemented this indication, although the level of evidence is still low.

### Treatment of hyperthyroidisms and thyrotoxicosis in pregnancy

Evidence is even more limited in pregnant women. According to the European Food Safety Authority, chronic exposure to perchlorates is not associated with teratogenic effects or other mechanisms of embryo and fetal toxicity, as similarly observed in adults as long as chronic consumption by food is lower than 0.3 μg/kg of body weight.

Chronic exposure to perchlorates by food, even contaminated food, is a rare event. However, consumers of exclusively plant-based diets may be exposed to this rare event, especially in the case of inadequate iodine supplementation. The risk of acute intoxication by perchlorate is negligible (https://www.efsa.europa.eu/en/efsajournal/pub/3869).

Nevertheless, the long-term effects of chronic exposure to perchlorate for therapeutical purposes are still unclear in human pathology. It is a partially positive result, especially in pregnant women, as lithium carbonate (another medication of the ionic inhibitor class) has been demonstrated to provide teratogenic effects, and its use in pregnancy is hence outlawed [33].

Sodium perchlorate is known to cross the haemato-placental barrier. However, a daily dose not exceeding 1000 mg per day of sodium perchlorate is not associated with adverse events as long as it does not lead to maternal hypothyroidism, thus putting off the potential adverse effects on fetal outcomes to iatrogenic hypothyroidism in the early stages of pregnancy rather than the exposure to perchlorate per se [3].

### Treatment of thyroid storm

The thyroid storm is a rare but potentially fatal condition. Only a few studies have been conducted to test the efficacy and safety of specific pharmacological treatments in thyroid storm with confusing results [34]. The literature review shows anecdotal cases and sporadic experiences [35–37] or case series [38] managed in emergency circumstances during intensive care with severe cases of thyrotoxicosis accompanied by hemodynamic decompensation and marked alteration of the level of consciousness (coma). High-dose thionamides are the treatment of choice. Ionic inhibitors, including perchlorates, may be considered an additional therapeutic option in patients requiring intensification of therapy as an add-on to methimazole, glucocorticoids, and beta-blockers.

## Conclusion

Ionic inhibitors are not considered the first-line treatment of hyperthyroidism due to several aspects related to efficacy/effectiveness, safety, and durability. Nevertheless, the ionic inhibitors have a rapid onset of action and can be considered in clinical cases requiring prompt management of severe manifestation (thyrotoxicosis, thyroid storm) or short-term and self-limiting conditions (AIT, iodine media contrast-induced hyperthyroidism, preparation to thyroid surgery) to avoid overexposure, and related risks, to methimazole. Less frequently, perchlorates are considered an alternative medical option for patients for whom antithyroid medications are not tolerated or contraindicated. In these cases, alternative strategies include radioactive iodine and thyroid surgery.

The first trimester of pregnancy could be another clinical context in which ionic inhibitors may be considered, even if antithyroid medications (especially propylthiouracil) are the first-choice option. Nevertheless, thionamides should be avoided in lactating women as these medications are typically excreted in maternal milk and could be associated with an increased risk of neonatal/infant hypothyroidism and intellective/motorial disorders.

The evidence level currently available from randomized and non-randomized observational studies assessing the efficacy/effectiveness and safety of perchlorate alone or combined with other medications is scanty. Besides methodological limits attributable to study design or conduction, the lack of specific outcomes (e.g., cardiovascular safety, mortality) is the leading limitation of the examined papers.

## Abbreviation

ATA: American Thyroid Association
ETA: European Thyroid Association
AIT: Amiodarone-Induced hyperThyroidisms
TSH: Thyrotropin
fT4: Free Thyroxine
fT3: Free Triiodothyronine
NIS: Sodium(Natrium)-Iodine Symporter
